# Hyperthermic intraperitoneal chemotherapy in management of malignant intraductal papillary mucinous neoplasm with peritoneal dissemination: Case report

**DOI:** 10.1016/j.ijscr.2019.09.033

**Published:** 2019-09-24

**Authors:** Chayanit Sirisai, Yutaka Yonemura, Haruaki Ishibashi, Satoshi Wakama, Akiyoshi Mizumoto

**Affiliations:** a NPO to Support Peritoneal Surface Malignanacy Treatment, Kyoto, Japan; b Peritoneal Dissemination Center, Kishiwada Tokushukai Hospital, 4-27-1 Kamoricho, Kishiwada, Osaka, 596-0042, Japan; c Department of Surgery, National Cancer Institute of Thailand, 268/1 RamaVI Road, Rajvithi, Bangkok, 10400, Thailand; d Department of Surgery, Kusatsu General Hospital, 1660 Yabasecho, Kusatsu, Shiga Prefecture, 525-8585, Japan

**Keywords:** IPMN, intraductal papillary mucinous neoplasm, PMP, pseudomyxoma peritonei, CRS, cytoreductive surgery, HIPEC, hyperthermic intraperitoneal chemotherapy, MPD, main pancreatic duct, CDDP, cisplatin, MMC, mitomycin C, 5-FU, 5 flourouracil, L-OHP, oxaliplatin, CPT-11, irinotecan, EUS-FNA, endoscopic ultrasonography with fine needle aspiration, CCR, completeness of cytoreduction, Pancreatic cystic tumor, Intraductal papillary mucinous neoplasm, Pseudomyxoma peritonei, HIPEC, Cytoreductive surgery, Peritoneal dissemination

## Abstract

**Introduction:**

Cytoreductive surgery (CRS) and hyperthermic intraperitoneal chemotherapy (HIPEC) are treatment to deal with peritoneal dissemination that has excellent result for various cancer especially pseudomyxoma peritonei (PMP), mesothelioma. For now, this combination of treatment is still running for pancreatic adenocarcinoma but no description for pancreatic cystic tumor like malignant Intraductal papillary mucinous neoplasm (IPMN).

**Presentation of cases:**

We report 3 cases of malignant IPMN with peritoneal dissemination that treated with CRS and HIPEC. Two cases have atypical presentation of malignant IPMN with PMP. HIPEC was performed using cisplatin-based regimen. The longest survival in this study is 93 months, compared to the median survival of 44.3 months without HIPEC.

**Discussion:**

CRS and HIPEC have not proven to treat in patient with pancreatic cystic tumor with peritoneal dissemination. But these treatments can be improved survival outcome in selected patient.

**Conclusion:**

CRS and HIPEC trend to improve survival. More studies need, not only to evaluate the role of HIPEC on malignant IPMN, but also prognosis and outcome.

## Highlights

• IPMN is not a common disease, has low risk to develop invasive lesion. But when invasion occurred, the prognosis is poor.
• Malignant IPMN with peritoneal dissemination also has poor prognosis.
• CRS and HIPEC can prolong survival in malignant IPMN with peritoneal dissemination patient.
• Pseudomyxoma peritonei can be found as rare manifestation of IPMN, that can associate with tumor rupture form diagnostic procedure like EUS-FNA or surgical procedure.

## 1 Introduction

Peritoneal dissemination has been regarded as end stage of disease but after introduction of locoregional therapy, Cytoreductive surgery (CRS) and hyperthermic intraperitoneal chemotherapy (HIPEC), has changed of this view. CRS and HIPEC has proved its benefit on Pseudomyxoma peritonei (PMP), mesothelioma, gastrointestinal tract cancer, and ovarian cancer. PMP, the presence of mucin in peritoneal cavity, originated from IPMN is rare condition, that was introduced by Zanelli et al. in 1998 [1]. IPMN is an uncommon mucin producing tumor and slow progressive disease that can present in wide spectrum of malignancy, range from benign to invasive behavior. Although risk of developing invasive tumor is low but once invasion is detected, the prognosis is poor [2].

We found peritoneal dissemination from malignant IPMN 3 cases in past 10 years. All cases underwent CRS and HIPEC under the same concept of other cancers.

## 2 Material and method

We reviewed medical records from 2008 to 2018, there were 3 cases of malignant IPMN with peritoneal dissemination. We have consent for the publication all three patients and accompanying images. The procedure-specific consent, patient data and material of this study had reviewed and approved by our institutional review board with ethic number H19, 2008. All patients underwent the same standard CRS procedure by surgical oncologist. HIPEC was discussed with patient and decided before procedure but if intraoperative bleeding more than 4000 ml, HIPEC will not performed. This work has been reported in line with the SCARE criteria. [3]

## 3 Presentation of cases

### 3.1 Case 1

A 69-year-old man presented with chronic pancreatitis. Computer Tomography (CT) showed cystic lesion at tail of pancreas 10 cm with mucinous ascites and scallop edge of liver and spleen. The appendix was unremarkable. Exploratory laparotomy was performed and found mucinous ascites without malignant cell. Large cystic mass at tail of pancreas with enlarged 10 mm. Cystic disruption was detected. Complete CRS was performed. Immediately after resection, HIPEC was administered with cisplatin (CDDP) 100 mg and mitomycin C (MMC) 20 mg in 42 °C condition for 60 min. Histology showed mucinous carcinoma on top of IPMN, MPD margin was not free from tumor but no lymphatic or perineural involvement was seen. Mucinous tumor was invaded to submucosa layer of stomach, right colon and to splenic parenchyma. No recurrence disease found during follow up period.

After 6 years of surgery, he had intestinal obstruction from recurrent tumor at pancreatic stump around previous ileo-colostomy anastomosis. Second operation was performed but tumors at subhepatic area and heptoduodenal ligament were intentionally left untouched due to thick adhesion. Completeness of cytoreduction (CCR) score was 3. HIPEC was repeated using oxaliplatin (L-OHP) 100 mg and 5-fluorouracil (5-FU) under hyperthermic condition (42 °C) for 60 min. Pathological report showed recurrent of pancreatic mucinous cancer. Unfortunately, 3 months after second operation, tumor was increased in size around hepatic hilum and pancreatic stump. Percutaneous transhepatic biliary drainage was performed to relief jaundice followed by proton beam radiation 52 Gy for 26 times. Complete response was achieved but he passed away from tumor complication. He survived for 93 months after initial diagnosis of malignant IPMN.

### 3.2 Case 2

A previously healthy 54-year-old man was noted of having high CA 19-9 on screening program. CT abdomen showed 5 cm cystic lesion at tail of pancreas. EUS found multiple cystic lesions connect to 1.1 cm MPD and mural nodule. Malignant IPMN was diagnosed. Distal pancreatectomy was performed. Pathological study revealed malignant IPMN without lymphatic involvement, free resection margin and no residual tumor found. After operation oral S1 was stated as an adjuvant treatment for 6 courses. 6 months after operation, CA19-9 was increased. CT showed left lower abdominal mass 3 cm suspected of peritoneal metastasis. S1 100 mg per day was started again and continued for 28 days. He underwent laparotomy 4 weeks after chemotherapy (CMT). Intraoperative finding showed no ascites, but fluid washing was positive for malignant cell. PCI score was 4. Macroscopic tumor was completely resected with extended right colectomy, omentectomy and peritonectomy. Metastatic carcinoma from malignant IPMN was confirmed. Adjuvant CMT was given.

**Fig. 1:**
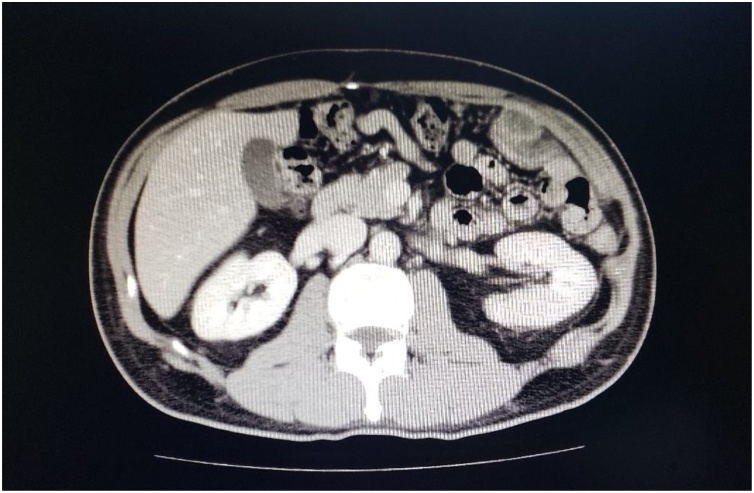
CT scan of the last follow up show no recurrent disease.

9 months after second operation, recurrent mass on the left abdominal wall was found. Re-exploratory laparotomy with CRS was performed and achieved CCR 0. HIPEC was administrated with MMC 20 mg and CDDP 40 mg at 42 °C for 40 min. Adjuvant CMT with irinotecan (CPT-11) and S1 was started. Now he survives well with chemotherapy session no evidence of disease recurrent on CT images (Fig. 1) during more than 33 months follow up.

### 3.3 Case 3

**Fig. 2:**
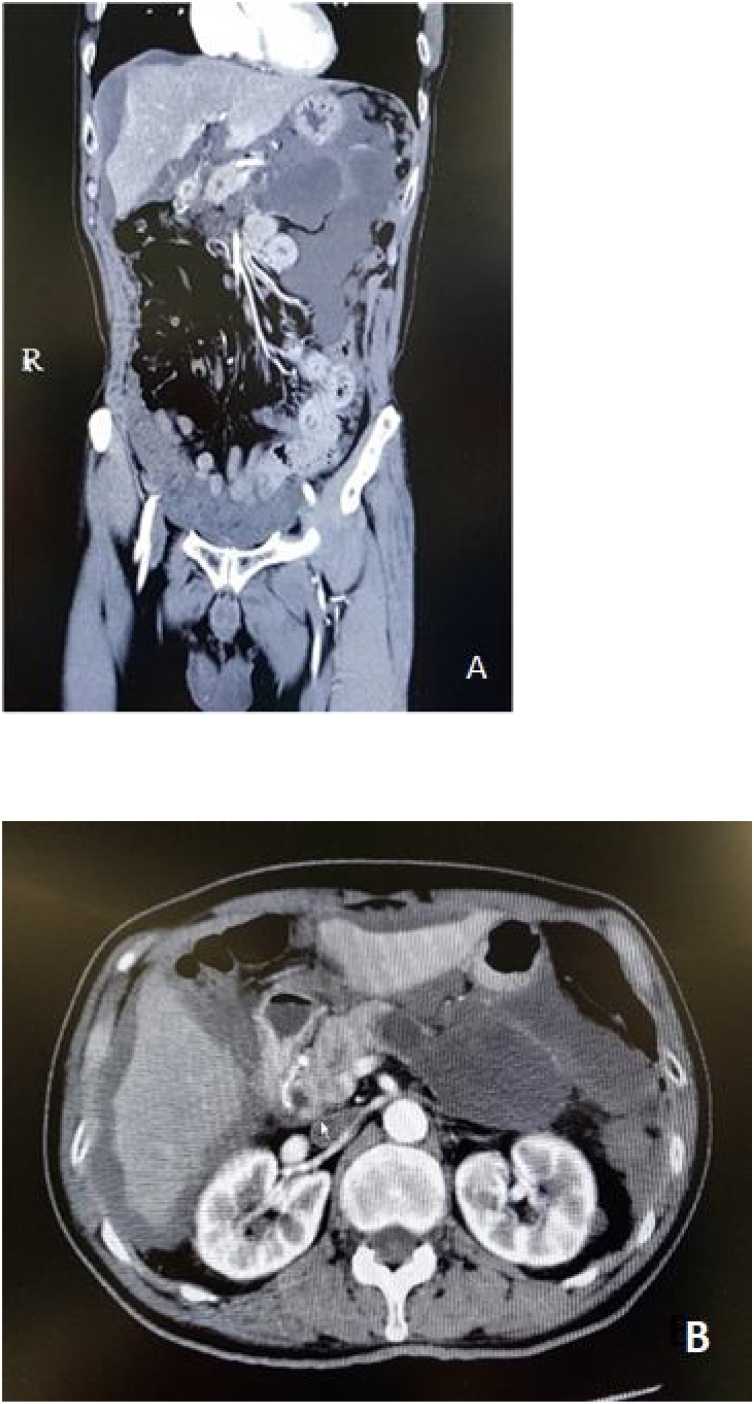
CT scan image showing cystic lesion at tail of pancreas and mucinous ascites (A) Coronal section show mucinous content around pancreatic cyst and upper abdominal part (B) Axial section show scallop liver.

A male, 69 years old with underlying of diabetes mellitus, hypertension and glaucoma. He came to the hospital with severe epigastric pain for 3 months which was spontaneously disappeared. Abdominal ultrasonography was periodically performed and revealed pancreatic cystic lesion at tail of pancreas size 3.1 cm in diameter. MPD was dilated 10 mm in diameter. No ascites, evidence of cystic disruption, mural nodule nor enhanced lesion was found in the subsequent EUS. 6 months later pancreatic cyst was increased in size to 6 cm, with disruption of pancreatic cyst and spreading of mucinous content in abdomen causing scallop edge of liver and spleen (Fig. 2). The diagnosis of IPMN with rupture pancreatic cyst and PMP was made. In the operative PCI was 23. Gelatinous content containing mild atypical cell was removed followed by distal pancreatectomy with splenectomy along with right hemicolectomy, total gastrectomy and peritonectomy. CCR 0 was achieved. Intraoperative bleeding was 4100 ml and HIPEC was omitted. Pathological study revealed malignant IPMN with rupture pancreatic cyst with high grade PMP, no lymphatic involvement and free margin. He survived more than 7 months after surgery with chemotherapy.

## 4 Discussion

IPMN is not a common disease but with recent increased in awareness. Many imaging modalities were introduced to improve lesion detection and differentiation of invasive foci. Prevalence of cancer in IPMN was 6–92% [2]. The role of cyst fluid analysis and cytology by endoscopic ultrasonography with fine needle aspiration (EUS-FNA) has the possibility of tumor cell spillage via needle tract into intraperitoneal cavity which can cause peritoneal dissemination [4]. Surgical resection has been recommended for IPMN with high risk stigmata (Obstructive jaundice in patient with a cystic lesion at head of pancreas, enhance mural nodule ≥5 mm, Main pancreatic duct size ≥10 mm) [5]. The malignant transformation occurs in 30%–60% of cases and turn out to be invasive cancer in 17%–43% [2,5]. IPMN with peritoneal metastasis has considered to be an end stage disease, without any current management guideline.

PMP, in literature reviewed, mostly associated with mucocele of appendix or ovarian mucinous tumor. PMP associated with pancreatic tumor was introduced by Zanelli et al. in 1998 [2]. Our study reported 2 cases of malignant IPMN with atypical presentation as PMP. Until now there have been only 11 cases reported, including this study, of PMP that associated with pancreatic cystic tumor [1,[6], [7], [8], [9], [10], [11]] show in Table 1. However, the mechanism of PMP from mucinous producing tumor of pancreas is unclear. We believed that main cause of mucinous ascites is the spillage of mucin into peritoneal cavity from rupture cyst or MDP, or fistula formation, and minor spillage from medical procedure, such as EUS-FNA or surgery. In our series, 2 of patients had the evidence of cystic wall disruption. From previous case report, one-third of patients have evidence of cystic disruption or fistula formation [9,[7], [8], [9], [10], [11]].

**Table 1:** Summary malignant IPMN case associated with PMP.

Reference	year	Age/sex	Pathology	Operation	Adjuvant treatment	Survival
Zanelli et al. [1]	1998	49/M	N/A	PD	–	>17 mo
Mizuta et al. [5]	2005	53/M	IPMC	Omentectomy and HIPEC	CDDP, etoposide, MMC/ IP GEM/ IV	>24 mo
Lee et al. [6]	2007	55/M	IPMC	PD	CCRT (Radiation + 5 FU)	>3 mo
Rosenberger et al. [7]	2007	75/M	MD	DP	None	>48 mo
Rosenberger et al. [7]	2007	75/M	IPMC	PPPD	RAD + CMT/IV	43 mo
Nepka et al. [10]	2009	82/M	N/A	None	None	>12 mo
Imaoka et al. [8]	2012	74/F	IPMC	None	N/A	42 mo
Imaoka et al. [8]	2012	56/M	IPMC	None	N/A	>48 mo
Arjona-Sanchez et al. [9]	2014	63/F	IPMC (recurrent)	Peritonectomy + CRS + HIPEC	MMC/ IP	>70 mo
Present case 1	2009	69/M	IPMC	DP + peritonectomy + CRS + HIPEC	GEM/IP + IV (1st) CDDP/IP (2nd), RAD	93 mo
Present case 3	2017	69/M	IPMC	DP + peritonectomy + CRS	–	>7mo^a^

PMP: Pseudomyxoma peritonei, N/A non-available, IPMC: Intraductal papillary mucinous carcinoma, MD: moderaty dysplasia, PD: Pancreaticoduodenectomy, DP: distal pancreatectomy, CRS: Cytoreductive surgery, HIPEC: Hyperthermic intraperitoneal chemotherapy, IP: intraperitoneal route, IV: Intravenous route, CDDP: Cisplatin, MMC: Mitomycin C, GEM: Gemcitabine, 5-FU: 5 flourouracil, L-OHP: Oxaliplatin, CPT-11: irinotecan CCRT: concurrent chemoradiation, RAD: radiaotherapy, CMT: chemotherapy.

a Disease free with chemotherapy.

Several treatment modalities have been utilized in management of peritoneal dissemination. Modern treatments such as peritonectomy and HIPEC, which were purposed by Sugarbaker PH [12], have been proved to improve of survival outcome in PMP which Five- and 10-year overall survival was 87.4% and 70.3% [4]. Not only in PMP, peritonectomy and HIPEC also showed benefit in GI tract cancer, ovarian cancer, and mesothelioma. In intraductal pancreatic adenocarcinoma (PDCA), peritonectomy and HIPEC increase 5-years survival rate from 15% to 23% and decrease incidence of local recurrence [13]. Nevertheless, there has been limited report for pancreatic cystic tumor. Theoretically, these cystic tumors have higher chance of accidental mucin spillage in to peritoneal cavity, causing spreading of malignant cells. As a result, direct intraperitoneal treatment might be one of the solutions to reduce recurrence with fewer side effects than systemic chemotherapy. In our series, the median survival time of 44.3 months (range 3–93 months) in patient who received CRS and HIPEC seems to be longer than 30.4 months (range 3–48 months) in other series [[6], [7], [8], [9], [10], [11]].

One case in this series had experienced intraoperative massive bleeding which is one of contraindication of HIPEC because hyperthermic condition will lead to vasodilatation and allow more bleeding from raw surface.

Recurrence rate of malignant IPMN was approximately 33.8% with median survival of 46 months [14]. On the other hand, no available data of recurrent malignant IPMN with peritoneal dissemination has been reported. There were 2 recurrence malignant IPMN in this series, received CRS and HIPEC, one can achieved CCR 0 and another one cannot (CCR 3). After operation both patients survived at least 17 months. Patient with IPMC and PMP have longest disease free about 93 months after diagnosis and disease-free survival is 72 months.

## 5 Conclusion

However, CRS and HIPEC are not yet approved for the treatment of pancreatic cancer. For malignant IPMN with peritoneal dissemination CRS and HIPEC can be successfully treated in selected case and trend to prolong survival outcome. Further studies are still needed to prove the real benefit of HIPEC on pancreatic cystic tumor.

## Sources of funding

This research did not receive any specific grant from funding agencies in the public, commercial, or not-for-profit sectors.

## Ethical approval

The procedure-specific consent, patient data and material of this study had reviewed and approved by the ethical review bodies of Kishiwada Tokushokai hospital with ethic number H19, 2008.

## Consent

We confirmed that we have consent for the publication of this case series for all three patients and accompanying images.

## Author’s contribution

Substantial contributions to conception and design by Y. Yonemura and C. Sirisai.

Acquisition of data by Y. Yonemura, H. Ishibahi, S. Wakama, A. Mizumoto.

Drafting the article by C. Sirisai.

Critical revision of manuscript for intellectual content by Y. Yonemura, A. Mizumoto.

All members have been approved the final version to be published.

## Registration of research studies

This study had been approved for registration at TCTR. The identification number is TCTR20190704001.

## Guarantor

Prof. Yutaka Yonemura.

Dr. Chayanit Sirisai.

## Provenance and peer review

Not commissioned, externally peer-reviewed.

## Declaration of Competing Interest

All authors declare no conflict of interest of any financial and personal relationship.
